# Long-lived laser-induced arc discharges for energy channeling applications

**DOI:** 10.1038/s41598-017-14054-z

**Published:** 2017-10-23

**Authors:** Guillaume Point, Leonid Arantchouk, Emmanuelle Thouin, Jérôme Carbonnel, André Mysyrowicz, Aurélien Houard

**Affiliations:** 1 Laboratoire d’Optique Appliquée, ENSTA ParisTech, CNRS, Ecole Polytechnique, Université Paris-Saclay, 828 boulevard des Maréchaux, 91762 Palaiseau cedex, France; 2 ONERA-CP, Chemin de la Hunière et des Joncherettes, 91123 Palaiseau cedex, France; 3 Laboratoire de Physique des Plasmas, CNRS, Ecole Polytechnique, Université Paris-Saclay, route de Saclay, 91128 Palaiseau cedex, France

## Abstract

Laser filamentation offers a promising way for the remote handling of large electrical power in the form of guided arc discharges. We here report that it is possible to increase by several orders of magnitude the lifetime of straight plasma channels from filamentation-guided sparks in atmospheric air. A 30 ms lifetime can be reached using a low-intensity, 100 mA current pulse. Stability of the plasma shape is maintained over such a timescale through a continuous Joule heating from the current. This paves the way for applications based on the generation of straight, long duration plasma channels, like virtual plasma antennas or contactless transfer of electric energy.

## Introduction

Laser filamentation is a nonlinear optical phenomena that was discovered in the early days of the laser era, when damage tracks and thin fluorescent channels were witnessed in transparent condensed matter through which a powerful laser pulse propagated^1,2^. But it is only after the chirped pulse amplification technique was devised^3^ that filamentation could be witnessed in gases^4^.

This propagation regime results from the dynamic competition between the self-induced collapse of a powerful laser pulse due to the optical Kerr effect, on the one hand, and diffraction, group velocity dispersion, nonlinear absorption of the laser energy and photoionization-induced plasma defocusing, on the other hand. This yields the formation of thin and long channels - or *filaments* - in which the pulse is able to maintain a very high intensity seemingly without suffering from diffraction^5–9^.

A very interesting property of filaments is that while propagating in this regime, a laser pulse deposits a significant part of its energy in the propagation medium over the whole filament length. This nonlinear energy absorption occurs chiefly through high-field ionization, but also from rotational stimulated Raman scattering^10^. After a nanosecond-scale thermalization, this fast energy deposition is eventually converted into heat and leads to the formation and subsequent hydrodynamic expansion of a hot air cylinder along the filament, leaving a central air channel with reduced density^11–17^. This in turn reduces the dielectric strength of air, meaning that a spark discharge can be induced at a voltage lower than the breakdown voltage by connecting two charged electrodes with filaments^12,18^. As such, laser filamentation was shown to be able to trigger and guide long sparks^18–21^, guide corona discharges^22^, and even deviate discharges from their natural path^23^. Recently, Clerici *et al*. also demonstrated control of curved spark trajectories by means of Airy laser beams instead of the widely used Gaussian beam^24^. Théberge and co-authors also showed that meter-sized, milliseconds duration guided discharges can successfully be developed using a double-circuit scheme^25,26^.

Filamentation-guided discharges have several interesting applications currently under active development. First, such straight and precisely controlled plasma columns can be used as a substitute for radio-frequency (RF) metallic antennas, sporting superior reconfigurability and stealth capabilities^27,28^. Laser-induced discharges have also long been envisioned as a means to transfer high intensity electrical energy. A filament lightning rod could be used to protect sensitive facilities by triggering the inception of downward leaders from lightning clouds^29^ or intercepting naturally-generated downward leaders before they reach the ground^23,30,31^. They can also be used to channel electrical energy along a well-controlled path, with several pending technologies like the design of sturdy and reliable closing switches with a very low jitter^32^ or even a replacement for the pantograph on trains, leading to the suppression of mechanical friction with overhead power lines^33,34^.

However several problems currently plague the development of such applications. Taking the example of the plasma antenna^28^, the performance of these technologies directly depends on the ability of the discharge plasma to remain at the required conductivity level for as long as possible. A second problem, which is expected to become more and more important as the plasma lifetime is increased, is to be able to preserve the shape of the plasma column for the required time.

We have previously shown that the temporal evolution of electron density in filamentation-guided discharges is dictated by the discharge current waveform when the current time evolution is slow^35^. From this finding we devised two different techniques to increase the plasma lifetime to a millisecond timescale, that we demonstrate in this Article:

- If a high electron density plasma is required, an oscillating current with high amplitude and low damping is best adapted. This scheme was tested using a homemade filament-triggered, 180 kV Marx generator^36^ to generate a 10 cm spark with ~10 μs lifetime before injecting ~100 A AC current in the resulting plasma to sustain it over a millisecond timescale.
- Conversely, when a lower electron density is required, a low-amplitude, long-lasting monopolar current pulse is the ideal solution. This scheme was tested using a very compact, strongly-damped 30 kV RC circuit delivering a ~10 ms, 100 mA current pulse over a 10 cm gap.

We also show that in both cases the discharge spatial shape can be preserved over such long timescales through the continuous Joule heating of the plasma by the residual current.

## Results

### Long duration, high current filamentation-guided discharges

**Figure 1:**
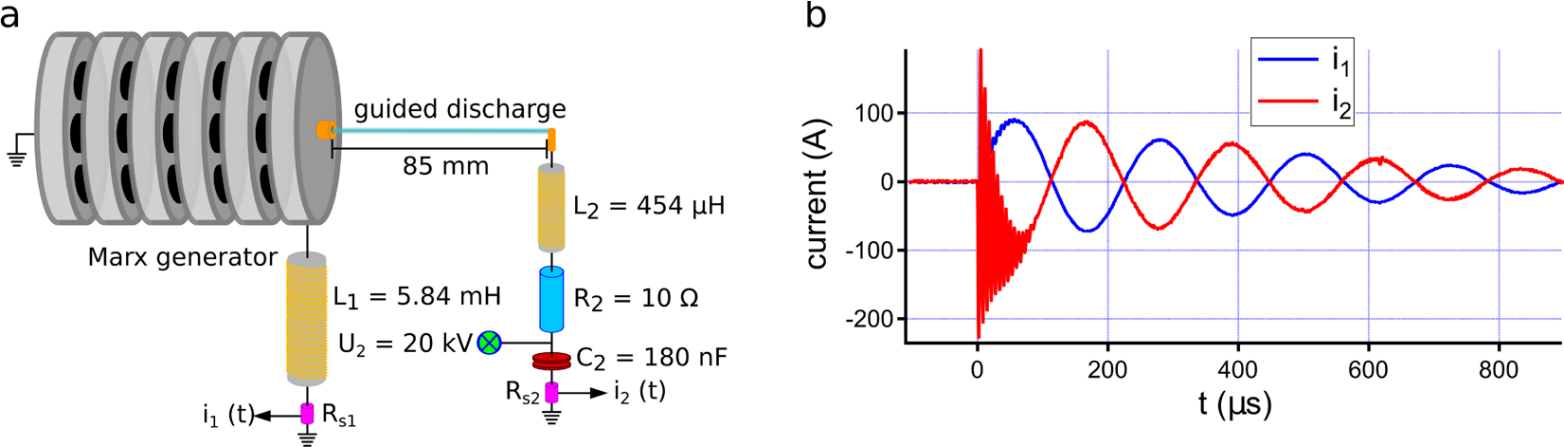
(**a**) Schematic description of the double circuit scheme. (**b**) Recorded waveforms for currents *i*_1_ and *i*_2_, clearly showing initial high-frequency and long term low-frequency oscillations.

The generation of millisecond-duration, 100 A sparks was done using a slightly modified version of the double-circuit scheme already used by Arantchouk *et al*.^25^. This scheme was built around a filamentation-triggered Marx generator as the high-voltage source. Using a single laser pulse, it has previously been demonstrated that it was possible to trigger both the generator, and a guided discharge at its output up to 21 cm long with a 180 kV output voltage^36^. In the present experiment the output voltage was reduced to 100 kV for practical purposes, while the gap was shortened to 85 mm to keep discharge jitter at a reasonable level. The double circuit is depicted in Fig. 1(a). It consists in two entangled RLC circuits. When gap breakdown occurs, the low energy (10 J) 5-stage Marx generator, starts to discharge in the first (R_2_L_2_C_2_) circuit provided that L_2_ ≪ L_1_. It results in damped sinusoidal oscillations at the high frequency *f*_*HF*_ = 156 kHz. Once the spark is established, the capacitor C_2_, initially charged to U_2_ = 20 kV for a 32 J total energy, starts to discharge through the plasma. By carefully choosing the value of L_1_ with respect to the Marx generator’s impedance, almost no return current goes to the generator. This secondary (R_2_L_1_L_2_C_2_) circuit is characterized by current oscillations with a much lower frequency *f*_*LF*_ = 4 kHz. By keeping the ballast resistor R_2_ at a small value (10 Ω), current damping is low enough so that it oscillates for more than 1 ms (see Fig. 1(b)). We can see that $|{i}_{1}(t)|\approx |{i}_{2}(t)|$ in this regime, showing the absence of return current in the Marx.

Guided discharges were characterized using three main diagnostics: electron and neutral air density profiles were recorded simultaneously by means of a two-color interferometer^37^. A high speed camera was used to record time-resolved pictures of the plasma emission in the visible range for the whole duration of a given discharge. Finally current viewing resistors (shunts) were used to record the time evolution of the discharge current.

**Figure 2:**
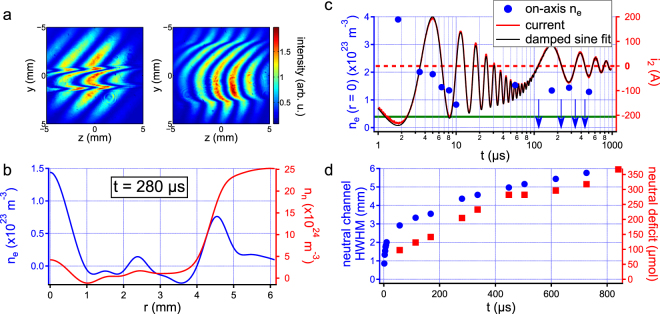
Two-color interferometry results on long-lived discharges. (**a**) Example of interferograms recorded at delay 2 μs (left) and 120 μs (right) in the single shot regime, showing the formation of a shock wave and the expansion of the subsequent channel. (**b**) Radial profiles of electron density (blue) and of neutral density (red) at delay 280 μs, showing the formation of a large channel with a strongly depleted neutral density while the plasma remains confined at the center in the form of a much narrower cylinder. Negative densities come from measurement uncertainties estimated at 4 × 10^22^ m^−3^ for electrons and 10^24^ m^−3^ for neutrals^37^. (**c**) Time evolution of on-axis electron density (blue circles) and of the discharge current (red solid curve). The electron detection threshold (green solid line) is estimated to be around 4 × 10^22^ m^−3^ ^37^. Electron density initially quickly decays, but starts oscillating with the low-frequency current at longer times. Blue arrows indicate times at which measurements could not detect free electrons. (**d**) Time evolution of the neutral channel radius (blue circles) and of the total quantity of lacking neutral molecules with respect to air in standard pressure and temperature conditions (red squares), showing the channel is still expanding and remains at a low density even after a delay 900 μs.

Results from the two-color interferometry are presented in Fig. 2. The simple study of interferograms (Fig. 2(a)) already brings useful information about the discharge physics, showing the formation of a steep optical index gradient propagating outwards at early times, leaving a perturbed cylinder at the center. This can be quite easily interpreted at the generation of a shock wave due to the sudden heating from the deposited energy in the plasma, which expels matter from the center of the discharge and leaves a tube of low density.

When looking at neutral and electron density radial profiles after the shock has left the field of view (Fig. 2(b)), one can see that free electrons are concentrated in the form of a narrow peak with a ~500 μm half-width at half-maximum (HWHM). A secondary ring is also observed at a radial position corresponding to the neutral channel boundary. However, one must be cautious about the reality of this peak at the edge of the low density channel, because its position corresponds to sharp changes in the interferograms and could therefore correspond to a measurement artifact. The maximum electron density reached is on the order of 10^23^ m^−3^. In the case of standard density air, this would correspond to a ionization ratio of less than 1%. However, one can see that the corresponding neutral profile shows a much larger channel with a ~4 mm HWHM and a quasi-depleted density. Given the resolution of our instrument on the order of 10^24^ m^−3^ ^37^, we therefore estimate that the residual air density in the underdense channel is below ~10% of the air density in normal pressure and temperature conditions *n*_0_ = 2.47 × 10^25^ m^−3^. Consequently the real ionization ratio of the discharge plasma is at least one order of magnitude higher than previously estimated. It is even possible that the central plasma column could actually be fully ionized.

Figure 2(c) shows the time evolution of on-axis electron density, compared to that of the discharge current. At early times, when the current evolves in the high frequency regime, the electron density decays relatively quickly, almost reaching the detection threshold by 10 μs. However at later times, in the low-frequency regime, it is back to a level ~10^23^ m^−3^. Interestingly, while the measurements performed at times corresponding to a local current extremum yield electron profiles, the electron density falls below the detection threshold when the current is near 0. It means that electron density starts to oscillate at twice the current frequency, as it was predicted in the case of a low frequency AC discharge current^35^.

Hint of this behavior was given by the dependence of plasma luminescence on the square of the current as recorded in reference^25,26^, but our measurement is the first to show a direct dependence of the plasma electron density on the discharge current in the AC regime. Therefore the design of specific current waveforms could enable one to precisely control the plasma density and reach even longer discharge lifetimes. Surprisingly the plasma resistance seems not to share the oscillatory behavior of electron density. Indeed discharge current is well fitted by a double damped sine:

1 $$\begin{array}{rcl}{i}_{2}(t) & = & {I}_{1}\,\sin \,\mathrm{(2}\pi {f}_{1}t+{\phi }_{1}){{\rm{e}}}^{(-t/{\tau }_{1})}\\  &  & +\,{I}_{2}\,\sin \,\mathrm{(2}\pi {f}_{2}t+{\phi }_{2}){{\rm{e}}}^{(-t/{\tau }_{2})},\end{array}$$

which corresponds to a double RLC circuit with constant component values (Fig. 2(c)). The low frequency oscillatory component decays with a characteristic time:

2 $${\tau }_{2}=2\frac{{L}_{1}+{L}_{2}}{{R}_{2}+{R}_{p}}=490\,{\rm{\mu }}s\mathrm{.}$$

In this precise case, neglecting the contribution of the contact resistance between the electrodes and the spark, we find the plasma resistance to be:

3 $${R}_{p}=15\,{\rm{\Omega }}\mathrm{.}$$

We can evaluate the plasma conductivity *σ*_*p*_ following:

4 $${\sigma }_{p}\approx \frac{{L}_{{\rm{gap}}}}{{R}_{p}\pi {\rm{\Delta }}{r}^{2}},$$

where *L*_gap_ is the discharge gap length and Δ*r* is the radius of the plasma equivalent conductor, taken as the electron density profile HWHM. Since the plasma radius was measured to be ~500 μm (Fig. 2(b)), *σ*_*p*_ is on the order of 10^4^ S · m^−1^. The pressure in the plasma is expected to be back to atmospheric pressure after a few hundred microseconds^38^. In this case temperature can be derived from plasma conductivity by computing transport coefficients of high temperature air^39^. This leads to a plasma temperature of ~13000 K. Since *p* ~ *p*_atm_ ≈ 10^5^ Pa, we can also estimate the air density in the plasma using the ideal gas law:

5 $${n}_{n}=\frac{p}{{k}_{B}T}\sim {10}^{23}\,{{\rm{m}}}^{-3}\mathrm{.}$$

As the electronic Debye length in the plasma is estimated at ~10 nm for an electron density of 10^23^ m^−3^ and a temperature of 10 kK, the discharge plasma must respect quasi-neutrality. Consequently, as the density of heavy species is estimated to be the same as electron density, this confirms our early assumption that the discharge plasma has an actual ionization ratio close to 1.

The oscillatory behavior observed with electron density is not shared by the neutral density profile (Fig. 2(d)). Indeed the underdense channel front keeps propagating in time, eventually reaching a 6 mm HWHM by 900 μs. Another precious information comes from the time evolution of the channel depth, here evaluated in terms of neutral molecule deficit over the whole discharge by integrating the density profile:

6 $${\rm{neutral}}\,{\rm{deficit}}=\frac{2\pi }{{{\mathscr{N}}}_{A}}{\int }_{0}^{{L}_{{\rm{gap}}}}{\int }_{{\mathbb{R}}}({n}_{0}-{n}_{n}(r,z))r\,{\rm{d}}r{\rm{d}}z,$$

where ${{\mathscr{N}}}_{A}$ is the Avogadro number. As seen on the graph, the depletion also keeps increasing. It means that the global hydrodynamic flux is still directed towards the outside of the channel at this time, driven by the continuous energy deposition from the Joule heating in the plasma. As long as this heating remains sufficient to displace neutrals outside of the discharge channel, hydrodynamic instabilities at the boundary are prevented from forming^40^. The discharge is therefore inherently stable.

**Figure 3:**
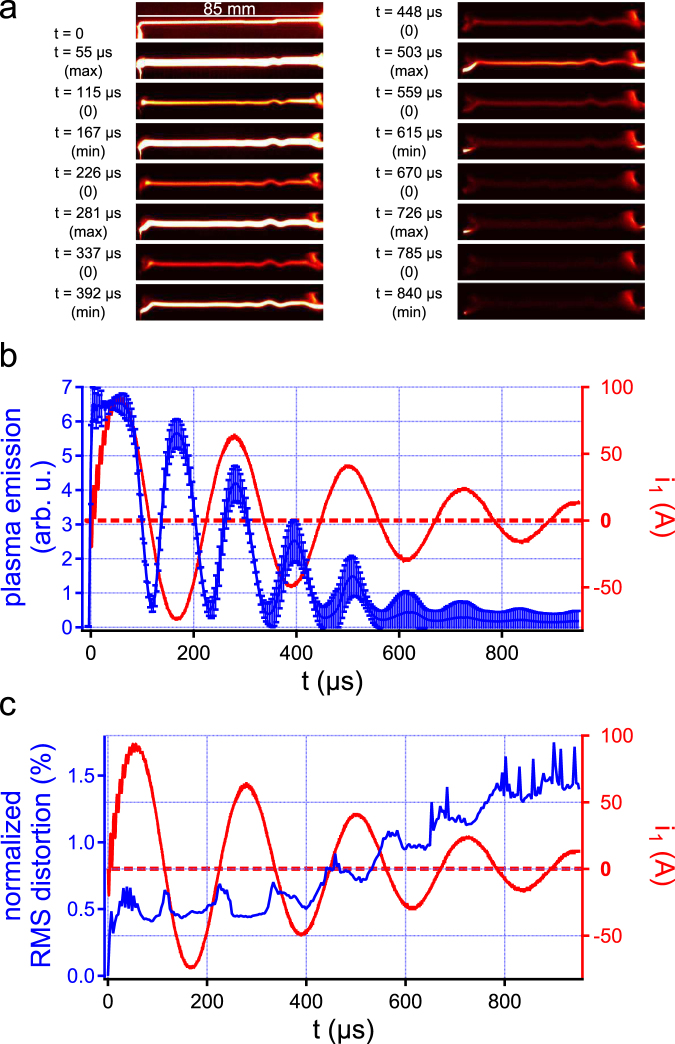
Imaging long-lived, high current discharges with a high speed camera. (**a**) False color sample images taken when the discharge current is either at a local extremum or at 0. (**b**) Time evolution of the spatial maximum of the plasma luminosity recorded by the camera, averaged over the discharge length. Error bars correspond to a confidence interval of ±1 standard deviation. (**c**) Time evolution of the normalized RMS distortion of the discharge plasma. The distortion slowly increases with time, experiencing sudden surges when the discharge current is near 0.

This analysis can be strengthened using results from the fast camera imaging of the discharge. This study was performed by recording images with a 288 kHz camera repetition rate and a 293 ns exposure time. As displayed in Fig. 3(b), the plasma luminescence evolves strongly in time, experiencing oscillations at nearly twice the discharge current frequency, an effect already identified in previous studies^25,26^. More importantly, as seen on the discharge pictures (Fig. 3(a)), the shape of the discharge remains stable with only marginal distortion throughout the whole discharge life. To better quantify this parameter, we make use of the normalized root mean square distortion (nRMSD), defined in the Methods section. A null nRMSD corresponds to a discharge keeping the same path for the whole discharge duration, while a nRMSD of 1 corresponds to a discharge where the amplitude of transverse distortions is on the order of the gap length. As seen in Fig. 3(c), the nRMSD of guided discharges remains at a very low level for the whole duration of the discharge on the order of 1%. However, it steadily increases in time in a fashion similar to that of the current envelope, and experiences small surges when the current approaches 0. This confirms the fundamental influence of the discharge current on the discharge stability through the continuous Joule heating of the discharge channel.

### Long duration, low current filamentation-guided discharges

**Figure 4:**
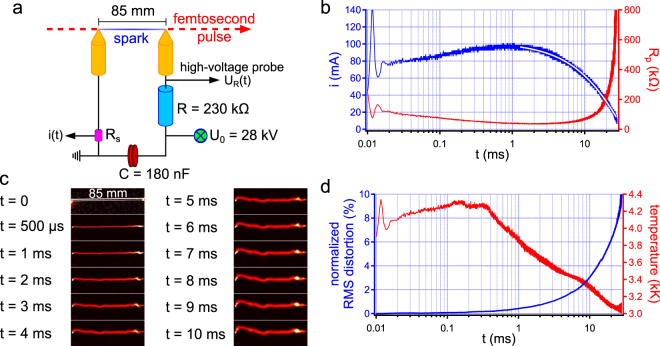
Long duration, low current guided discharges. (**a**) Schematic description of the experimental setup. (**b**) Time evolution of the measured discharge current (blue), which follows an exponential decay (white), and of the plasma resistance (red). (**c**) False color sample images of the discharge. (**d**) Time traces of the normalized RMS distortion (blue) and of the estimated plasma temperature (red), showing that the Joule heating of the plasma is not sufficient to prevent the distortion of the discharge at long times.

Long-lived, low current discharges were investigated using a much simpler setup than previously. It consists in a simple RC circuit (Fig. 4(a)), in which the capacitor is charged to a voltage U_0_ before filaments are used to close the gap and generate a guided discharge. The goal here was to initiate a very long current pulse with a low amplitude, leading us to use a very high ballast resistor of 230 kΩ. In order to extend the gap to 85 mm, like in the previous case, with a voltage as low as 28 kV, we make use of an axicon lens instead of a spherical lens to generate laser filaments, a technique which proved able to decrease the breakdown voltage of air by one order of magnitude^24^. This result represents an even higher increase in discharge length than was observed with AC high voltage (~400%)^41,42^.

Since insulation constraints are much lower than in the case of the Marx generator, it is possible to continuously record the gap voltage using a simple high-voltage probe. This enables us to track the time evolution of the plasma resistance. As seen in Fig. 4(b), the current pulse generated by the gap closure has the characteristic shape of an exponential-decaying pulse from a RC circuit with a very long duration of more than 30 ms and a low peak amplitude of 100 mA. Note that the total electric energy is equivalent to the one used with the double circuit (70 J vs. 42 J). The long current decay thus ensures that the low current pulse will result in a discharge with a much longer duration than with the Marx generator.

With such a simple setup, we therefore achieved a discharge duration one order of magnitude higher than the current record obtained for filamentation-triggered arcs^26^. The corresponding plasma resistance has a completely different behavior from that of long-lived, high current discharges. First, the order of magnitude of this parameter peaks at 100 kΩ, which is 10^4^ times higher than previously. Such a high resistance is very uncommon for discharges, still it does not prevent the plasma from reaching an unprecedented lifetime. Second, the resistance also strongly varies with time, reaching a steady state by 1 ms at ≈50 kΩ before sharply increasing at the end of the discharge, when plasma is starved of current, reaching 400 kΩ by 30 ms.

Compared to the Marx discharges, the current is ~10^2^ lower (100 mA versus ~10 A at long times) and the plasma resistance is ~10^4^ higher (100 kΩ versus ~10 Ω), meaning the Joule heating should stand at the same level in both cases. Therefore, as the discharge stability directly depends on this parameter as shown by Shneider^38^, plasma distortion in the case of low current discharges should remain at a level comparable to that of Marx discharges. Consequently we used the same diagnostic as before: films recorded using a fast camera. Figure 4(c) presents sample images of one such film, showing the discharge remains quite straight for a few milliseconds before irremediably distorting near the grounded electrode. Evaluating the normalized RMS distortion as in the previous section yields the curve displayed in Fig. 4(d). One can see that the nRMSD equals 2% after 5 ms, increasing linearly with time and increasing well above 10% at the end of the discharge. Looking at the previous results (Fig. 3(c)), the 2% level was reached by only 800 μs with the oscillating high current. A continuous Joule heating therefore appears more favorable than an alternating one to ensure the discharge stability. This can be assessed by estimating the time evolution of the plasma temperature as it was done in the previous section: after a few hundred microseconds the plasma is back to atmospheric pressure, meaning that the temperature can be unambiguously extracted from a measurement of the plasma conductivity, which in turn can be readily extracted from the plasma resistance and the plasma section. The result in plotted in Fig. 4(d): plasma temperature keeps increasing for ~300 μs up to 4200 K before smoothly decaying, eventually reaching 3000 K after 30 ms as the current is unable to sustain the Joule heating of the discharge. Inevitably this results in a loss of stability.

## Discussion

Two main facts can be assessed from these experiments: first, the discharge plasma from a previous, short duration spark can be sustained for a much longer time by channeling electric energy from another source. Second, this sustaining can be achieved using a very low current amplitude. The key parameter ensuring the discharge stability appears to be the plasma temperature. When the temperature decreases, a flow from the surrounding air will start to fill the low-density tube surrounding the plasma, eventually becoming turbulent and strongly disrupting the discharge straightness^38^. Conversely if the temperature can be maintained through the Joule heating of the plasma, this deleterious effect can be prevented.

Therefore the lengthening of the discharge lifetime is an aspect that can be completely dissociated from the lengthening of the discharge gap. Indeed already exist methods based on filamentation-guided discharges that can be used to generate meter-long sparks using small-scale high-voltage sources^41,42^. Once a well-guided spark is generated, only the external circuit supplying current to the plasma can play a role in the discharge stabilization. One has to be particularly careful to the current waveform used to make sure that the plasma temperature, and therefore the discharge stability, is maintained.

In these experiments we only used tabletop voltage sources and relatively small capacitors. With a larger power supply, one could extend the lifetime of a guided discharge by several orders of magnitude, at least until convection of the generated hot air finally disrupts the channel.

## Methods

### Guided discharge generation

The generation of guided discharges was done using a chirped pulse amplification Ti:sapphire laser chain able to deliver pulses as short as 50 fs and with an energy up to 200 mJ. In the case of the Marx generator, 200 mJ, 700 fs pulses were focused using a 5 m spherical lens, generating a ~2 m long multifilament bundle both in the discharge gap and through the whole Marx generator. For the low-current discharges, the spherical lens was replaced by an axicon lens with a 5° apex angle, yielding a much shorter plasma column with a ~20 cm length using a 100 mJ, 50 fs laser pulse. As both setup used gaps initially charged with a constant voltage, no synchronization was required between the laser system and the high-voltage circuits.

### Discharge diagnostics

- Films of discharges were made using a Fastcam SA-X2 ultrafast camera from Photron, Inc.
- The density of heavy species and of free electrons in the plasma was recorded using a two-color interferometer. This works by recording simultaneously the plasma refractive index at two different wavelengths. As the contribution of heavy species is almost independent of the wavelength, while it has a quadratic dependence for free electrons, one can then solve an equation system with two unknowns to finally retrieve plasma densities^37^. Interferometry was performed transversely on the Marx generator discharges, followed by Abel inversion of the recorded phase shift profiles, enabling us to extract free electron density and neutral density with a 1 cm wide field of view, a 10 μm spatial resolution and a 10 time resolution, yielding a ~10^22^ m^−3^ resolution for electron density and a ~10^24^ m^−3^ resolution for neutral density.
- For both low and high current discharges, current waveforms were recorded using current viewing resistors (shunts) from T & M Research Products.
- Gap voltage for low-current discharges was tracked using a PVM-1 high-voltage probe from North Star High Voltage.

### Discharge shape stability indicator

**Figure 5:**
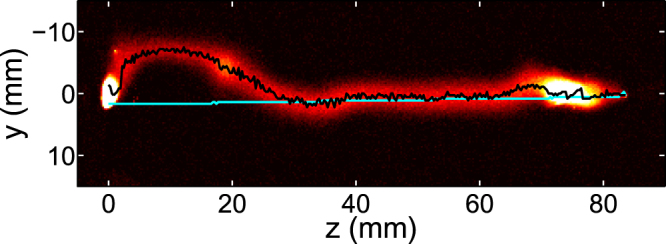
Definition of the root mean square distortion at time *t* from side pictures of discharges: ridge of the 2D luminescence profile (black) and reference curve (light blue), that is the ridge of the luminescence profile taken at *t* = 0.

We designed a scalar indicator to assess the shape stability of a guided discharge, based on time-resolved side pictures of the arc. These pictures can be described as two-dimensional (*y*, *z*) luminescence maps, where *z* is taken along the discharge axis (Fig. 5). For each picture, taken at different times, the curve yielding the position *y*_max_(*z*, *t*), that is the luminescence ridge position (black curve on Fig. 5), is extracted. This curve is to be compared to a reference curve *y*_ref_(*z*) (light blue curve on Fig. 5), which is taken as the luminescence ridge curve *y*_max_(*z*, *t* = 0) estimated at the breakdown time. The indicator, dubbed *root mean square distortion*, or RMSD, is calculated at time *t* following:

7 $${\rm{RMSD}}(t)=\sqrt{\frac{{\sum }_{i=1}^{{N}_{{\rm{pixels}}}}{({y}_{{\rm{\max }}}({z}_{i},t)-{y}_{{\rm{\max }}}({z}_{i},t=\mathrm{0)})}^{2}}{{N}_{{\rm{pixels}}}}},$$

*N*_pixels_ being the number of pixels along *z*.

The *normalized* RMSD, or nRMSD, is just taken as:

8 $${\rm{nRMSD}}(t)=\frac{{\rm{RMSD}}(t)}{{L}_{{\rm{gap}}}},$$

where *L*_gap_ is the length of the discharge gap along *z*. A null nRMSD means that the discharge has exactly the same shape as it has initially. A nRMSD equal to 1 means that the transverse distortions of the discharge with respect to the reference path are on the order of the gap length, a very high value.

### Data availability

The datasets generated and analyzed during the current study are available from the corresponding author on reasonable request.

## Conclusion

In this Article we investigated two different schemes for increasing the lifetime of laser filamentation-guided discharges based on the design of the discharge current waveform. In the first one, a first electrical circuit was used to generate a first spark, before a second circuit started to discharge through the resulting plasma column, sustaining it for more than 1 ms, three orders of magnitude longer than with the first circuit alone. In the second scheme, a single, low amplitude and long duration current pulse was used directly from the inception of the filament-triggered discharge, yielding an even longer arc of 40 ms. In both cases, the discharge stability was maintained for ~1 ms by means of Joule heating from the current itself, preventing the turbulent decay of the low-density tube surrounding the plasma column. Although these experiments were performed with relatively small-scale discharges (85 mm), they can be easily transposed on larger setups because the initial spark generation and the subsequent sustaining of the discharge are completely uncorrelated. In recent experiments we increased the gap length to the meter-scale without much difficulty. This will be reported later.

As such, these results represent a significant achievement for the development of technologies in need of a non-solid, long-lived and stable conductor like the virtual RF antenna or the contactless transfer of electrical energy.
